# Automated side-chain model building and sequence assignment by template matching

**DOI:** 10.1107/S0907444902018048

**Published:** 2002-12-20

**Authors:** Thomas C. Terwilliger

**Affiliations:** a Mail Stop M888, Los Alamos National Laboratory, Los Alamos, NM 87545, USA

**Keywords:** model building, template matching

## Abstract

An algorithm is described for automated building of side chains in an electron-density map once a main-chain model is built and for alignment of the protein sequence to the map. The procedure is based on a comparison of electron density at the expected side-chain positions with electron-density templates. The templates are constructed from average amino-acid side-chain densities in 574 refined protein structures. For each contiguous segment of main chain, a matrix with entries corresponding to an estimate of the probability that each of the 20 amino acids is located at each position of the main-chain model is obtained. The probability that this segment corresponds to each possible alignment with the sequence of the protein is estimated using a Bayesian approach and high-confidence matches are kept. Once side-chain identities are determined, the most probable rotamer for each side chain is built into the model. The automated procedure has been implemented in the *RESOLVE* software. Combined with automated main-chain model building, the procedure produces a preliminary model suitable for refinement and extension by an experienced crystallographer.

## Abstract 2

A method for automated macromolecular side-chain model building and for aligning the sequence to the map is described.

## 1. Introduction   

Building side chains of an atomic model into an electron-density map is a very different problem to building the main chain. Normally the main chain is built first, so the approximate location of the C^β^ atom of the side chain and the approximate direction of the C^α^—C^β^ and C^α^—C bonds are already known by the time side chains are built. On the other hand, the identities of the side chains at each position in the map are normally not known at this stage, and at moderate resolution (∼2–3 Å) in a map of moderate quality it can be very difficult to distinguish many of the side chains. Consequently, the bulk of the problem is not placing the side chains but rather identifying them.

A number of methods have been developed to address the problem of identifying and fitting side-chain density. Most of the methods use the electron density at the coordinates of atoms of a side-chain model to evaluate the model. Jones *et al.* (1991 ▶) used a rotamer library (Ponder & Richards, 1987 ▶) to assist in manual fitting of side chains. Oldfield (2002 ▶) and Levitt (2001 ▶) fit a rotamer libraries to side-chain density, considering density at the positions of the atomic coordinates. In contrast, Morris *et al.* (2002 ▶) directly build a side-chain model from the coordinates of free atoms representing peaks in the density. In another very different approach, Holton *et al.* (2000 ▶) used machine-learning techniques to identify side chains in a map using methods that are rotation-independent, so that they require C^α^ positions but not the directions of the C^α^—C^β^ or C^α^—C bonds; the fitting of side chains to the density is then carried out by maximizing the correlation of density with that from a side-chain template. Pavelcik *et al.* (2002 ▶) described a method for template matching of arbitrary fragments of structure to a map by a rotation/translation search that could be used to identify side chains without knowledge of the main-chain coordinates.

Here, we describe a method for side-chain identification that uses the correlation of side-chain rotamer templates with density in the map to evaluate the fit of a side chain to the map. The side-chain templates are built from average density in refined models so that they reflect the patterns of density found in real structures. A Bayesian approach is used to estimate the probability that each side chain is present at each site. These probabilities are then used to align the protein sequence to the main-chain tracing of the map.

## 2. Methods   

### 2.1. Side-chain rotamer library   

Libraries of side-chain rotamers have been constructed numerous times (Ponder & Richards, 1987 ▶; Dunbrack & Cohen, 1997 ▶; Lovell *et al.*, 2000 ▶) and it would be possible to use one of these as the basis for modeling side chains. However, for the present purposes it was necessary to have a paired rotamer library and corresponding averaged template density library, so it was most convenient to construct both libraries at once from the same database. A library of side-chain rotamers was constructed using 574 refined protein structures chosen arbitrarily from non-redundant PDB files (Berman *et al.*, 2000 ▶; Hobohm *et al.*, 1993 ▶) with *R* factors of 20% or lower and a resolution of 1.8 Å or better. In order to limit the total number of rotamers for all amino acids, the maximum number of rotamers considered for any one amino acid was 40. For each amino-acid position in these refined protein structures, the coordinates of the main-chain N, C^α^ and C atoms were used to place the side chain in a standard orientation. Then, for each amino-acid type, all conformations of the amino-acid side chain in this group of structures were listed. A library was generated consisting of the smallest subset of these conformations that could be found such that every conformation in the list differed from a member of the library by at most 0.8 Å r.m.s. For several amino acids (methionine, lysine, glutamine, glutamate, asparagine and tyrosine) this was not possible with just 40 rotamers in the library and in these cases some configurations are not represented. Additionally, for arginine the N^η1^ and N^η2^ atoms were not included in the r.m.s.d. calculation for defining the libraries; even so, more than 40 rotamers would have been required to represent all configurations found and the list was truncated at 40 rotamers. A total of 503 side-chain rotamers were present in the entire library of side chains.

### 2.2. Side-chain rotamer electron-density templates   

The library of side-chain rotamers was used to cluster all conformations of each side chain from the 574 refined protein structures into rotamer groups. These groups contained from one to 18 543 conformations. For each rotamer group, a template was then constructed from the average electron density for the entire group, calculated using the coordinates and thermal factors from the refined structures (mapped into the standard orientation). In this way, the electron-density templates reflected both the variability in side-chain conformation within a rotamer group and the pattern of thermal factors from atom to atom in a rotamer. The electron-density templates were sampled on a grid with a spacing of 1 Å. All points within 3 Å of an atom in a side chain in one or more of the conformations present in the corresponding rotamer group were contained in the templates and all other points excluded. The templates for different amino acids and for different rotamers were therefore sampled at partially overlapping sets of lattice points. The region defining the template for glycine is not well defined by these criteria and as a special case the template for glycine was calculated in the same region as the template for alanine.

### 2.3. Estimation of the side-chain probability   

The relative probability that each of the 20 amino-acid side chains was located at a side-chain position in a polypeptide chain was estimated in several steps. The overall strategy at each position in the chain was to find the rotamer of each side chain that fitted the density best, then to use the fits of these 20 side chains to the density to obtain the probability for each possible side chain at that position.

Firstly, the correlation coefficient cc_*jk*_ of each side-chain rotamer density template *j* with the electron density at each side-chain position *k* in the polypeptide chain was determined. This was accomplished after transforming the density in the map to the standard reference frame defined by the main-chain N, C^α^ and C positions. For each side-chain type, only the best-fitting rotamer was considered further.

Next, a *Z* score was calculated for the fit of each side-chain template to each side-chain position. The *Z* score was based on the correlation cc_*jk*_ for the fit and the correlations for the fits of this template *j* to all other side-chain positions. The *Z* score calculated in this way describes how likely it is that the value of the correlation cc_*jk*_ would be obtained by chance. This was used to estimate the probability of measuring a value cc_*jk*_ for a template that is incorrect. We further assumed that the correct template can have any value of the correlation. This is a useful assumption because although we expect that a correct template will have a high value of the correlation, it is difficult to specify how high this value should be.

Using these assumptions, the mean correlation for the template *j* to all side-chain positions, 〈cc_*j*_〉, was used as an estimate of the correlation to be expected for this side chain to arbitrary side-chain density (*i.e.* generally not associated with this rotamer). Similarly, the standard deviation of this correlation, 〈σ_*j*_〉, was used as an estimate of the variation of this correlation to be expected for arbitrary side chains. Then the *Z* score, *Z*_*jk*_ = (cc_*jk*_ − 〈cc_*j*_〉)/σ_*j*_, was expected to be related to the probability of obtaining this value of the correlation by chance (that is, if side chain *j* is *not* the correct side chain at position *k*) using the relation *p*(cc_*jk*_) ≃ exp(−*Z* 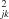/2). In order to prevent very poor fits from being confused with very good ones, *p*(cc_*jk*_) was taken to be unity for values of *Z* < 0 in this calculation.

Finally, the probability that amino-acid type *i* is the correct one at position *k* was calculated from Bayes’ rule. The *a priori* probability for each amino acid *p*_*oj*_ was estimated from numbers of each amino acid *j* in the protein, *n*_*j*_. The probability of observing the set of correlations {cc_*jk*_} at position *k* if the correct amino acid at this position is *i* is the product over all of the other amino-acid types *j* (not including the correct one, *i*) of the probabilities of observing the correlations {cc_*jk*_} by chance,
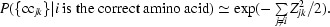
Using Bayes’ rule, we then estimate the probability, *p*_*ik*_, that amino acid *i* is the correct one at position *k*, yielding, after some simplification,
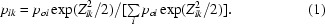



### 2.4. Alignment of fragments of main-chain model to the protein sequence   

A fragment of main-chain model containing *n* residues was matched to the protein sequence using the matrix of probability estimates *p*_*ik*_ describing the probability that amino acid *i* is the correct type at position *k* in the model. As the main-chain model might sometimes be missing amino acids (commonly at loop positions) or might cross from one segment of chain to another incorrectly, a first step in the alignment was to identify the sub-fragment of the fragment which could be matched to the sequence with the highest probability. This was considered likely to be the longest segment that is contiguous in the protein chain. The match of this sub-fragment was identified and the remainder of the fragment was then considered independently as a separate fragment.

To accomplish the identification of a contiguous sub-fragment and its match to the sequence, the alignment procedure described next was carried out *n*(*n* − 1)/2 times, once for each possible sub-fragment of the *n*-residue model fragment. Two different scoring algorithms were used in this process. For comparisons between sub-fragments of the same length, the probability of the alignment of each sub-fragment was used as the score. For comparisons between sub-fragments of different lengths, the probability estimates were not a good indicator of the relative qualities of the alignments and instead the score for each was 〈*Z*〉*N* ^1/2^, where *N* is the number of residues in the sub-fragment and 〈*Z*〉 is the mean *Z* score 〈*Z*_*jk*_〉 of the amino acids in this alignment.

For a given sub-fragment or fragment with *m* residues, all possible alignments *l* of the model with *m* sequential amino acids in the protein were considered. The relative probability *p*_*l*_ that alignment *l*, with amino-acid type *t*_*k*_ matched to position *k* in the model, was correct was estimated assuming that the probability estimates for all the residues in the model were independent, leading to
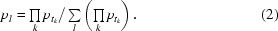
The sequence alignment was considered to be reliably identified if the probability for one match was at least 95% (that is, the combined probability of all other matches was 5% or less). Once a reliable sequence alignment was identified, the amino-acid sequence was mapped onto the model fragment. At each residue, the most probable rotamer corresponding to the amino acid assigned to that residue was built into the model.

## 3. Results and discussion   

The reliability of the probabilistic method described here for side-chain assignment and sequence alignment was examined using the density-modified electron-density map for eight different experimental maps with varying resolution, quality (figure of merit) and number of residues in the asymmetric unit. The main chain was built into each map as described in Terwilliger (2003 ▶). The coordinates of the main-chain atoms were then used to place side chains.

**Figure 1:**
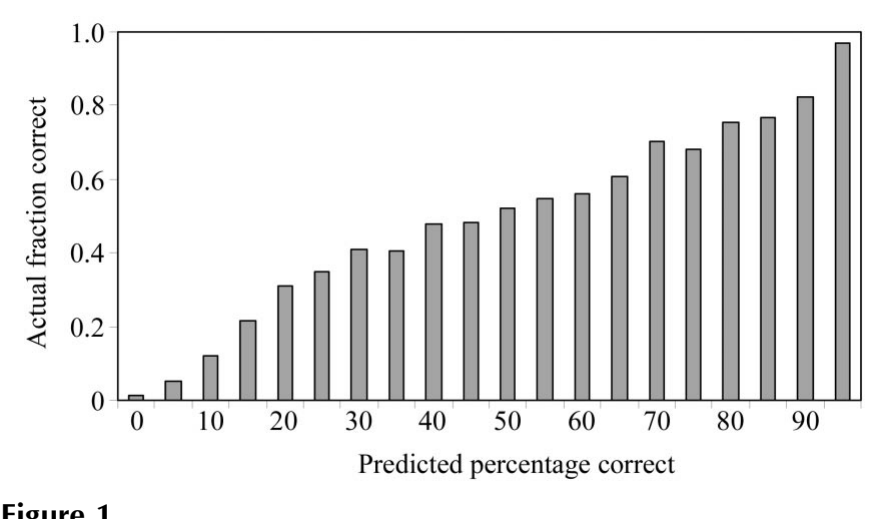
Fraction of correct amino-acid side-chain assignments as a function of the probability estimated from (1). For each residue in the main-chain models for the eight structures listed in Table 1 ▶, the relative probabilities for each of the possible side chains were obtained using (1). The correct side chains were identified as the nearest amino acid in the refined model of each structure. The fraction of correct amino-acid side-chain assignments is tabulated as a function of the probability estimates.

We first evaluated the utility of (1) by determining how well it actually predicts the probability that a particular side chain is present at a particular position in the model. Fig. 1 ▶ shows a histogram of the fraction of correct amino-acid side-chain assignments as a function of the probability assessed using (1 ▶). A total of 4349 side-chain densities from the eight experimental electron-density maps were compared with 20 side-chain templates to generate the histogram. The correct side chain at each position was identified from the refined model of the corresponding protein. Overall, Fig. 1 ▶ shows that the probability estimates obtained from (1) give a very good indication of the actual probability that the assignments are correct for these test cases.

**Figure 2:**
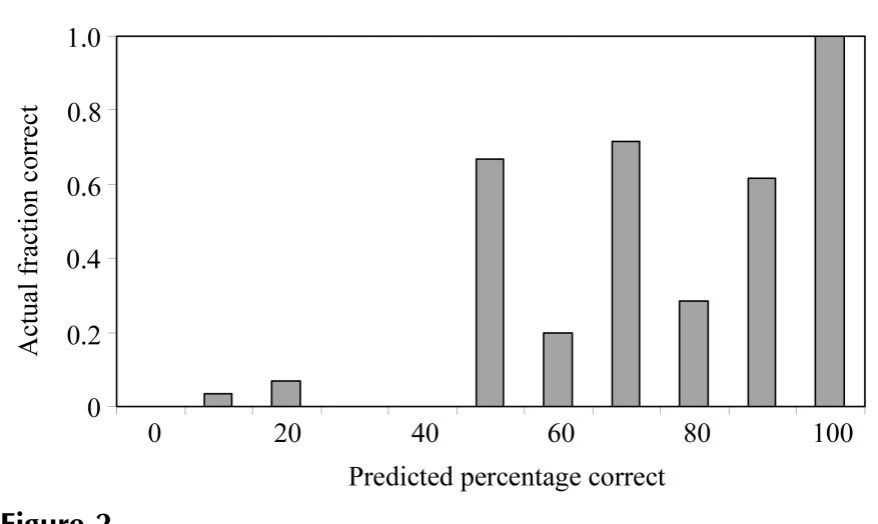
Fraction of correct fragment alignments as functions of the probabilities estimated from (2). For each main-chain fragment built, the sub-fragment with the highest weighted *Z* score was identified as described in §[Sec sec2]2. All alignments of this sub-fragment with the protein sequence were considered and the relative probabilities of each alignment were estimated with (2). An alignment was considered correct if the residue numbers of 90% of the residues in the fragment matched those of the nearest amino acid in the refined model.

We next evaluated how well the sequence-alignment probabilities estimated with (2) relate to the actual probabilities. Fig. 2 ▶ shows a histogram of the fraction of correct sequence alignments as a function of the probabilities of correct assignments estimated using (2). Assignments were considered correct if 90% or more of the residues in the alignment match the closest residue in the refined structure. A total of 85 very strong predictions with confidence >97% were obtained and all but one of these were correct assignments. Overall, the probability estimates for sequence alignment using (2) are fairly accurate, though there is less of a clear discrimination among alignments with moderate probability than in the case of the side-chain assignments.

The overall side-chain modeling results are summarized in Table 1 ▶. The side-chain modeling procedure identified and aligned the protein sequence to 71% of the 5131 residues in eight proteins. These eight proteins included two that were used in the development of the method (NDP kinase and gene 5 protein). They also included two that were among those in the database of proteins used to construct side-chain templates (gene 5 protein, PDB code 1vqb; β-catenin, PDB code 1dow). These three may therefore be slightly better fitted than a typical protein. The remaining five are likely to be relatively good indicators of the utility of the method. For the set of eight proteins as a whole, 99% of the sequence assignments were correct. The mean difference in coordinates between the side-chain atoms of the model and those of the corresponding refined structures was 1.3 Å and the r.m.s. difference was 1.8 Å, including lysine and arginine residues, where the positions of atoms in even the refined structures is often somewhat uncertain, but excluding atoms more than 10 Å away from any atom in the refined structures.

**Figure 3:**
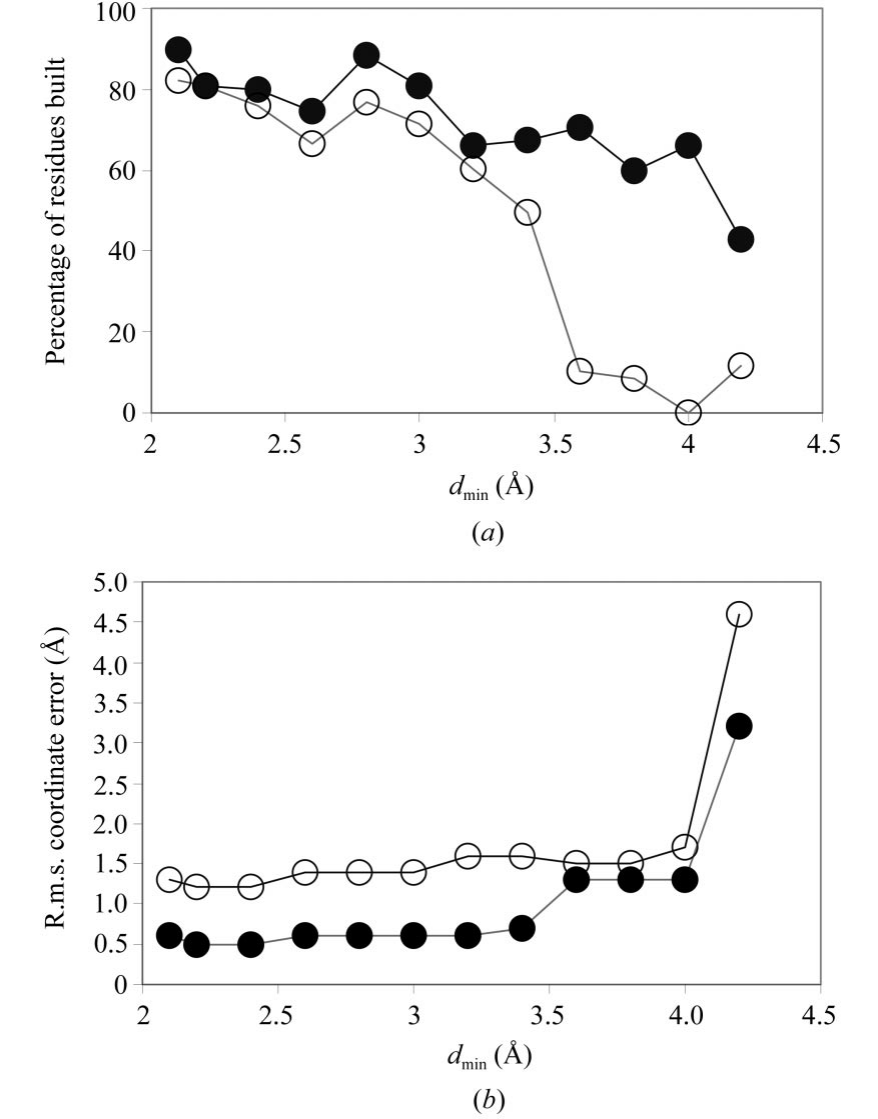
Effect of resolution on model building of IF5A. The phases and amplitudes for IF5A (Peat *et al.*, 1998 ▶) after density modification were truncated at varying resolutions and the resulting maps were used for automated main-chain and side-chain model building. (*a*) Percentage of the main chain (closed circles) and the side chains (open circles) in the refined structure that were built. (*b*) R.m.s. coordinate error for main-chain (closed circles) and side-chain (open circles) atoms. Side-chain atoms include C^β^.

To test the resolution-dependence of side-chain model building, the IF5A structure was built at a variety of resolutions, truncating the data in each case. This of course does not fully simulate the ability to build a model for a poorly diffracting crystal, as the data and phases are very good to the resolution cutoff in this test. Nevertheless, it can give an idea of what is possible with very good data. Fig. 3 ▶(*a*) shows the number of main-chain residues and side chains built as a function of the high-resolution cutoff. Fig. 3 ▶(*b*) shows the r.m.s. coordinate error in main-chain and side-chain atoms for the same models. Fig. 3 ▶ illustrates that in the presence of very good data, as much as 75% of the main chain and 50% of the side chains can be built at a resolution as low as 3.4 Å and with an r.m.s. coordinate error that is only slightly higher than at a resolution of 2.1 Å.

## 4. Conclusions   

The probabilistic methods described here for identifying side chains and their rotamers in the electron density at positions derived from a main-chain tracing are found to be very effective. With a map of reasonable quality and a segment of ten residues or longer, the alignment to the sequence can often be identified with a confidence greater than 98%.

As a side effect of aligning the model to the sequence, the quality of the main-chain protein tracing can be considerably improved. This is largely owing to the identification of errors in the main chain and elimination of the corresponding segments of main-chain model. For example, mis-tracings caused by tracing the chain through a loop region using fewer residues than are present in the actual loop are removed in this procedure. The procedure of finding the longest sub-fragment of a main-chain fragment that had a very strong match to the protein sequence was very useful for identifying these cases. This removal of residues is the reason for the differences between the number of main-chain residues in the models in this work compared with those obtained when using only the main-chain tracing algorithm (Terwilliger, 2003 ▶).

The algorithm for side-chain fitting and alignment developed here does have significant limitations. The side-chain rotamer libraries used are not as complete or as accurate as others available (*e.g.* Dunbrack & Cohen, 1997 ▶; Lovell *et al.*, 2000 ▶). This means that not all reasonable rotamers in proteins can be accurately represented by one of those in the library and that some side chains will therefore be poorly fitted. This could be improved by using a more complete library, but at a cost of examining a larger number of templates for a fit to the electron-density map. A possible compromise would be to use a more complete library only for those side chains that are not well fitted with a rotamer from the standard one. It could also be improved by using a filtered library such as that of Lovell *et al.* (2000 ▶), which removes conformations that are unlikely to be correct, or by explicitly checking side chains for poor contacts. An additional limitation is that some common situations are not recognized by the rotamer libraries (and by the main-chain model building that precedes side-chain fitting). These include disulfide bonds in proteins, unusual amino acids and all non-protein electron density. In most of these situations, the model simply does not include the corresponding region. There could be cases where such density is misinterpreted in terms of main-chain and side-chain conformations that are in the corresponding libraries, however.

## Figures and Tables

**Table 1:** Test structures for which side-chain models have been built with *RESOLVE*

Structure	Resolution ()	Figure of merit *m*	Residues in refined model	Main chain built (%)	Side chains built (%)	Correct alignment (%)	Side-chain mean coordinate error ()	Side-chain r.m.s. coordinate error ()
Gene 5 protein (Skinner *et al.*, 1994 ▶)	2.6	0.62	87	61	11	100	1.2	1.4
Granulocyte-stimulating factor (Rozwarski *et al.*, 1996 ▶)	3.5	0.70	242	50	0	N/A	0	0
Initiation factor 5A (Peat *et al.*, 1998 ▶)	2.1	0.85	136	84	84	99	1.3	1.8
-Catenin (Huber *et al.*, 1997 ▶)	2.7	0.72	455	81	62	100	1.2	1.7
NDP kinase (Pdelacq *et al.*, 2002 ▶)	2.6	0.56	556 (3 186)	56	37	98	1.2	1.6
Hypothetical (*P. aerophilum* ORF, NCBI accession No. AAL64711; Fitz-Gibbon *et al.*, 2002 ▶)	2.6	0.58	494 (2 247)	79	75	98	1.3	2.0
Red fluorescent protein (Yarbrough *et al.*, 2001 ▶)	2.5	0.91	936 (4 234)	88	88	99	1.2	1.8
2-Aminoethylphosphonate (AEP) transaminase (Chen *et al.*, 2000 ▶)	2.6	0.84	2232 (6 372)	85	81	99	1.3	1.8
